# The Specificity of Anticipatory Competence in Adolescents with Intellectual Disabilities

**DOI:** 10.1192/j.eurpsy.2026.11255

**Published:** 2026-07-31

**Authors:** A. Akhmetzyanova, T. Artemyeva, U. Zhirnova

**Affiliations:** Department of Psychology and Pedagogy of Special Education, Kazan Federal University, Kazan, Russian Federation

## Abstract

**Introduction:**

Anticipatory competence is a personality trait that serves as an important component in the regulation of activity, behavior, and emotional responses. The level of development of anticipatory abilities directly affects an adolescent’s adaptation to the surrounding environment. The capacity for forecasting constitutes a key resource for the successful socialization of adolescents with intellectual disabilities.

**Objectives:**

the study of the specificity of anticipatory competence in adolescents with intellectual disabilities.

**Methods:**

The study involved 51 adolescents aged 12 to 16 years (6A00.0, ICD-11) who were attending an educational institution for children with disabilities. The research was conducted using the following methods: Anticipatory Competence Test by V. D. Mendelevich; Study of Anticipatory Competence in Adolescents by A. I. Akhmetzyanova and T. V. Artemyeva; Test of Propensity for Deviant Behavior by E. V. Leus and A. G. Solovyov; and Propensity for Deviant Behavior by A. N. Orel.

**Results:**

The results of the study revealed that the personal-situational, spatial, and temporal components of anticipatory competence are below normative levels, which indicates a pronounced anticipatory deficit. Adolescents with intellectual disabilities were also found to have a situational predisposition toward unlawful behavior and a predisposition toward self-injurious behavior. From an early age, adolescents with intellectual disabilities experience difficulties in interacting with society, which is largely due to their having a limited social circle restricted to family members and a few peers. This is associated with difficulties in accurately assessing interpersonal situations and understanding the emotional reactions of others. An insufficient or weakened capacity for self-regulation in interpersonal interactions reduces the effectiveness of selecting adequate coping strategies, weakens self-control over one’s actions, and complicates the correct perception and comprehension of the current situation.

**Conclusions:**

for the successful overcoming of the identified difficulties, adolescents with intellectual disabilities require comprehensive support from specialists. One of the key tasks of such specialists should be the development of anticipatory competence and self-regulation skills, which contribute to successful socialization and reduce the risk of deviant behavior. This study was carried out with the support of the grant ‘Digital Socialization of Adolescents with Intellectual Disabilities’, aimed at conducting fundamental and exploratory research in scientific and educational institutions, enterprises, and organizations of the real sector of the economy of the Republic of Tatarstan.

**Disclosure of Interest:**

None Declared

